# Factor IX p.A37V mutation causes severe bleeding in a patient with phenprocoumon therapy

**DOI:** 10.1186/s40001-021-00533-7

**Published:** 2021-06-29

**Authors:** Nils Mülling, Vivian Rosery, H. Christian Reinhardt, Maher Hanoun

**Affiliations:** 1 Department of Nephrology, University Hospital Essen, University Duisburg-Essen, Hufelandstr. 55, 45147 Essen, Germany; 2 Clinic for Internal Medicine (Tumor Research), University Hospital Essen, University Duisburg-Essen, Hufelandstr. 55, 45147 Essen, Germany; 3 Department of Hematology and Stem Cell Transplantation, University Hospital Essen, University Duisburg-Essen, Hufelandstr. 55, 45147 Essen, Germany

**Keywords:** Vitamin K antagonists, Bleeding, Acquired hemophilia, Factor IX mutation

## Abstract

**Background:**

Bleeding is the most common complication of oral anticoagulants, due to inadequate dosing.

**Case presentation:**

This report describes the clinical course of a patient who developed severe bleeding under therapy with phenprocoumon, despite an INR in the lower therapeutic range. Strikingly, aPTT was prolonged, while factor IX activity was significantly reduced. Acquired hemophilia was excluded, due to missing detection of inhibitors. Finally, sequencing part of the factor IX gene including nucleotide position c.110 revealed a hemizygous factor IX mutation c.110C > T p (Ala37Val).

**Conclusions:**

In rare cases, missense mutations in factor IX propeptide are associated with severe bleeding complications. The substitution of alanin at position 37 to either valin or threonin (Ala37Val or Ala37Thr) leads to hypersensitivity to vitamin k antagonists.

## Background

Bleeding is the most common complication of oral anticoagulants, due to inadequate dosing. This report describes the clinical course of a patient who developed severe bleeding under therapy with phenprocoumon, despite an INR in the lower therapeutic range. Strikingly, aPTT was prolonged, while factor IX activity was significantly reduced. Acquired hemophilia was excluded, due to missing detection of inhibitors. In rare cases, missense mutations in factor IX propeptide are associated with severe bleeding complications [1]. The substitution of alanin at position 37 to either valin or threonin (Ala37Val or Ala37Thr) leads to hypersensitivity to vitamin k antagonists [1, 2].

## Case presentation

**Fig. 1:**
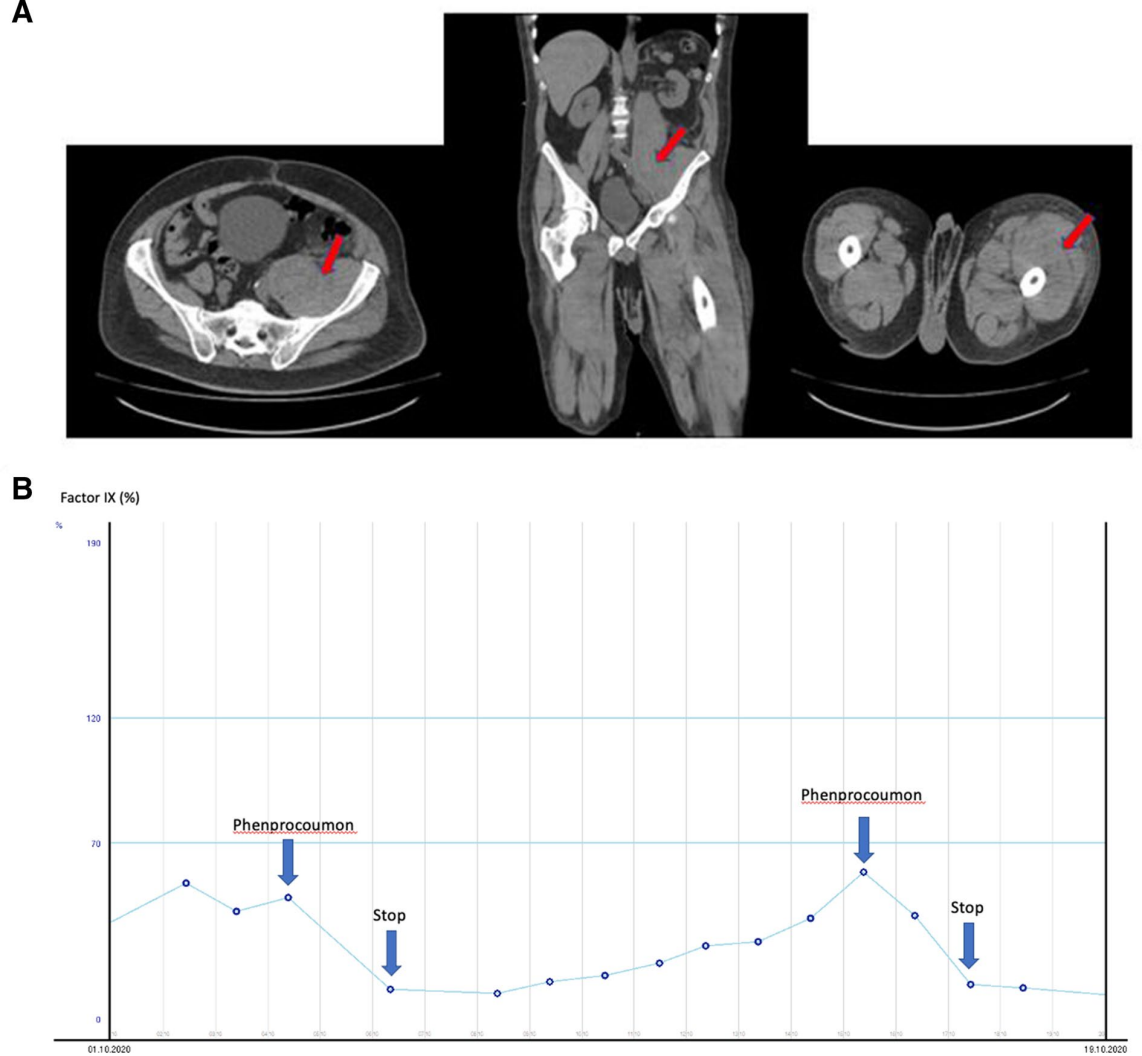
**A** CT images (native investigation) demonstrating severe hemorrhage into left psoas major, iliacus muscle and left thigh, indicated by the red arrows. **B** Factor IX activity during phenprocoumon rechallenge. “Stop” indicates discontinuation of treatment

A 55-year-old male patient was admitted to an external hospital with deterioration in general condition and dyspnea. A few months earlier, the patient underwent mechanical aortic valve replacement, due to infective endocarditis and hereafter received phenprocoumon. During the inpatient stay, hemoglobin levels dropped from 10.2 to 3.9 g/dl. While INR was with 2.29 in lower therapeutic range, aPTT was significantly increased reaching 104 s (Table 1). Computed tomography showed pronounced retroperitoneal hemorrhage. After repeated bleeding and surgical interventions, the patient was referred to our department. Here, he presented with spontaneous bleeding into the left psoas major, iliacus muscle and the left thigh (Fig. 1A). Phenprocoumon was paused and substituted by unfractionated heparin. After detecting a substantially reduced factor IX activity of 3%, mixing studies were performed, showing a gradual normalization of factor IX activity, two hours after incubation with increasing doses of normal plasma. Together with missing detection of inhibitors, acquired hemophilia could be ruled out. After cessation of phenprocoumon and application of unfractionated heparin, factor IX activity recovered gradually within approximately 10 days. The patient then resumed treatment with phenprocoumon. However, within few days factor IX activity rapidly dropped and this was reproduced in a second approach (Fig. 1B). Finally, sequencing part of the factor IX gene, including nucleotide position c.110 revealed a hemizygous factor IX mutation c.110C > T p (Ala37Val). Anticoagulation has subsequently been continued with low molecular weight heparin under close monitoring of anti-factor Xa activity and regular cardiovascular examinations. Factor IX activity remained within normal range, no further relevant bleeding or thromboembolic events occurred. Replacing the mechanical aortic valve with a biological valve is still under evaluation.

**Table 1:** aPTT, thrombocytes, prothrombin time, INR and activities of the measured coagulation factors at time of the bleeding

Parameter	Result	Normal range
Thrombocytes (10^3^/µl)	439	163–337
Prothrombin time (%)^a^	34	70–130
INR	2.29	0.8–1.2
aPTT (s)	104	23.6–34.8
Factor VIII (%)	246	80–216
Factor IX (%)	3	78–150
Factor XI (%)	111	83–154
Factor XII (%)	72	> 53
Factor XIII (%)	78	> 86

^a^Prothrombin time expressed as percent according to Quick

## Discussion and conclusions

Bleeding is a major complication of treatment with phenprocoumon in case of overdose [3]. Bleeding complications with isolated prolongation of aPTT are suggestive for acquired hemophilia, which is induced by inhibitory autoantibodies [4]. Mixing studies confirm the diagnosis, as a small proportion of normal plasma compensates for clotting factor deficiency and normalizes clotting time. However, in the presence of inhibitors, normalization does not occur even with higher doses of normal plasma [4]. The gene encoding for factor IX is located on the long arm of the X chromosome (Xq27.1). Two missense mutations in the factor IX propeptide coding region at the Ala-10 residue have been described for an exchange of alanine (GCC) to valine (GCT) or threonine (ACC) [1]. In our case, the Ala-10 [GCC] to Val [GCT] mutation was detected. These mutations lead to hypersensitivity of factor IX to vitamin K antagonists as the -10 position of the factor IX propeptide contains the binding site for γ-carboxylase. An amino acid exchange at this point leads to significantly reduced binding affinity of factor IX for γ-carboxylase. Under normal conditions, this induces a slight reduction in factor IX activity without clinical relevance. However, in case of oral anticoagulation with vitamin K antagonists, lack of biologically active vitamin K combined with reduced affinity of factor IX propeptide for γ-carboxylase leads to a clinically relevant decrease in factor IX activity with high risk of bleeding [5]. There is only imprecise information on the prevalence of factor IX germline mutations. Previously, the examination of 255 unrelated X-chromosomes for the presence of factor IX mutation did not reveal any case. The authors estimated a prevalence below 1.46% at 95% confidence interval [1]. Ulrich et al. extrapolated the prevalence in Switzerland at 1: 10,000 to 1: 100,000 [2]. On top, only rare cases of severe bleeding events in patients carrying factor IX mutation have been reported [1, 2, 5, 6].

This report highlights that aPTT should be determined in patients with an unclear bleeding event under therapy with vitamin K antagonists. In cases of a significantly prolonged aPTT, factor IX mutations should be excluded [2].

## Data Availability

Not applicable.
